# The effect of different grazing conditions on the insulin and incretin response to the oral glucose test in ponies

**DOI:** 10.1186/s12917-019-2088-1

**Published:** 2019-10-16

**Authors:** Danielle M. Fitzgerald, Christopher C. Pollitt, Donald M. Walsh, Martin N. Sillence, Melody A. de Laat

**Affiliations:** 1 School of Earth, Environmental and Biological Sciences, Queensland University of Technology, Brisbane, QLD Australia; 2 School of Veterinary Science, University of Queensland, Gatton, QLD Australia; 3 Animal Health Foundation, Pacific, MO USA

**Keywords:** Equine metabolic syndrome, Fertiliser, Horse, Hyperinsulinaemia, Insulin dysregulation, Laminitis, Pasture

## Abstract

**Background:**

The oral glucose test (OGT) is a useful tool for diagnosing insulin dysregulation (ID) and is somewhat repeatable in ponies under consistent management. This study aimed to determine whether the insulin and incretin responses to an OGT in ponies differed after short-term access to fertilised pasture, compared to unfertilised pasture, by using a randomised, repeated measures study design.

Sixteen mixed-breed ponies were classified as severely insulin-dysregulated (SD; post-prandial insulin ≥80 μIU/mL) or not severely insulin-dysregulated (NSD; post-prandial insulin < 80 μIU/mL) using an OGT prior to the study. The ponies accessed pasture that was fertilised, or unfertilised, for 5 days (4 h/day, with supplemental hay provided at 0.7% bodyweight), with a 10 day period between phases. An OGT was performed after each phase. Glucose, insulin, active glucagon-like peptide-1 (aGLP-1), and glucose-dependent insulinotropic polypeptide (GIP) were measured in post-prandial blood samples.

**Results:**

The volume of fertilised pasture was five-fold greater than unfertilised pasture, with % non-structural carbohydrates (NSC) similar between all forages. Consuming fertilised pasture increased (*P* = 0.018) the serum insulin response to an OGT, compared to grazing unfertilised pasture. A limitation of the study was that pasture intake was unable to be quantified. Insulin responses were greater in SD, compared to NSD, ponies (*P* < 0.001) and remained well above the test cut-off at all times. A subset of ponies, initially screened as NSD, became (more) insulin-dysregulated after pasture access. Further, aGLP-1 was a significant predictor of insulin concentration in this cohort.

**Conclusions:**

Whereas some insulin-dysregulated ponies were comparatively resistant to dietary intervention, others showed markedly different OGT responses following subtle changes in their forage-based diet. This implies that mild/early ID might be unmasked by dietary change, and that dietary management is important in these ponies. However, dietary management alone may not be adequate for all cases of ID.

## Background

Pasture-associated endocrinopathic laminitis is common [1], and occurs most frequently in spring and summer [2], suggesting that particular pasture conditions may exacerbate endocrine dysfunction and increase the risk of laminitis [3, 4]. Insulin dysregulation (ID) is the most important risk factor for laminitis, and post-prandial hyperinsulinaemia occurs in at-risk animals after the consumption of non-structural carbohydrates (NSC) [5–7]. Pasture can be high in NSC, and immoderate NSC intake while grazing is likely to exacerbate ID.

Despite the risk of laminitis in some animals, horses are grazers and pasture will remain a popular forage for horses and ponies [8–10]. In order to maintain pastures and provide an adequate (or superior) plane of nutrition, fertilisation has been utilised to increase pasture growth for many grazing species, including *E. caballus* [10, 11]. Many studies have shown that although fertilised pasture has an accelerated growth rate resulting in increased pasture availability, the NSC content of fertilised pastures may decrease due to the increased energy required for rapid growth [12, 13]. Increased hyperinsulinaemia (without a decrease in insulin sensitivity) has been observed in ponies fed a high NSC diet [14], and insulin sensitivity decreased when horses were fed a diet high in starch/NSC [15–17]. However, no studies have specifically examined the effect of grazing pasture on the degree of ID in horses or ponies.

Currently, the oral glucose test (OGT) is an appropriate preliminary test for investigating ID as it accounts for small intestinal glucose absorption, hepatic glucose uptake, pancreatic function and the enteroinsular axis, where incretin hormones derived from the small intestine augment the pancreatic insulin response to glucose [18–21]. The main incretin hormone known to play a role in equine ID is active glucagon-like peptide-1 (aGLP-1), with a second incretin, glucose-dependent insulinotropic peptide (GIP), also potentially of importance [21–23]. The OGT involves in-feed administration of dextrose, with subsequent measurement of the post-prandial circulating insulin concentration. It has been reported to be repeatable in ponies fed a consistent diet [19], and has been able to predict laminitis occurrence in ponies fed a high NSC diet [24].

The aim of this study was to determine whether the insulin and incretin responses to an OGT in ponies differed after short-term access to fertilised pasture, compared to unfertilised pasture. A secondary aim was to determine whether the post-prandial response to the OGT differed after pasture access in ponies with varying degrees of insulin regulation (i.e. not severely insulin dysregulated (NSD), compared to severely insulin-dysregulated (SD) ponies).

## Results

### Animals

The sixteen ponies (eight mares and eight geldings, 13 ± 6 years) were above ideal body condition (BCS; 6 [1.75], CNS; 2.5 [1.75]), but were otherwise healthy and were not lame during the study. Based on the results of the initial OGT (Table 1), ten ponies were classified as NSD, and six were SD. Although there was no difference between the groups in the blood glucose concentration before the OGT test meal, SD ponies had higher post-prandial glucose (Table 1). Serum insulin concentrations were increased in the SD group both before (4-fold, *P* = 0.028) and after (13-fold, *P* = 0.002; Table 1) the meal, compared to the NSD group. There were no differences in body weight or BCS between the groups. However, CNS was higher in SD ponies, compared to NSD ponies (*P* = 0.019; Table 1).

**Table 1:** Morphometric measurements and diagnostic OGT parameters in groups of ponies with and without severe insulin dysregulation

	NSD	SD	*P* value
Age	13 (7)	12 (4)	0.597
Bodyweight	222 [260]	164.5 [64]	0.356
BCS	6.5 [3.2]	6 [1.2]	0.654
CNS	2 [1.5]	3 [0]	0.019
0 h Resting Glucose	4.9 [0.55]	5 [2.5]	0.586
2 h Post-prandial Glucose	6.6 (0.9)	7.7 (1.1)	0.049
0 h Resting Insulin	2 [2.1]	7.3 [18.8]	0.028
2 h Post-prandial Insulin	23.4 [45.1]	298 [121]	0.002

Key: Data are mean (± sd) or median [IQR], *BCS* body condition score, *CNS* cresty neck score, *NSD* not severely insulin-dysregulated (*n* = 10), *SD* severely insulin-dysregulated (*n* = 6)

### Diet

The mean NSC content (%; starch plus water soluble carbohydrates) of the hay, and the fertilised and unfertilised pasture, was similar (Table 2). Compared to unfertilised pasture, the fertilised pasture had greater sward height (10.6 ± 0 .1 cm vs 4.3 ± 0.3 cm; *P* = 0.003) and sward density (58.5 ± 0.1 g/10cm^2^ vs. 28.8 ± 1.8 g/10cm^2^; *P* = 0.001). Thus, the volume of fertilised pasture in a given area was five times greater than that of unfertilised pasture. The maximum ambient daily temperature during the month of the study ranged from 27 to 36 °C, with a total monthly rainfall of 72.7 mm.

**Table 2:** Forage analyses

	Units	LOD	Hay	UF (Phase 1)	F (Phase 1)	UF (Phase 2)	F (Phase 2)	*P* value
DM field sample	%		–	43.6	39.7	43.3	41	0.04
Sward height	cm		–	4.65	10.5	3.98	10.68	0.003
Sward density	g/10cm^2^		–	27.5	58.5	30	58.5	0.002
WSC	%	4.0	4.3	4.9	< 4.0	9.2	6.9	0.5
Starch	%	2	3	3	2	4	4	0.7
NSC	%		7.3	7.9	6	13.2	10.9	0.5

Key: All results are reported on a dry matter basis. All units of % are g/100 g equivalent. *LOD* limit of detection, *UF* unfertilised pasture, *F* fertilised pasture, *DM* dry matter, *WSC* water soluble carbohydrate, *NSC* non-structural carbohydrate calculated as WSC + starch

### Hormone responses to the oral glucose tests

#### The whole cohort

**Fig. 1:**
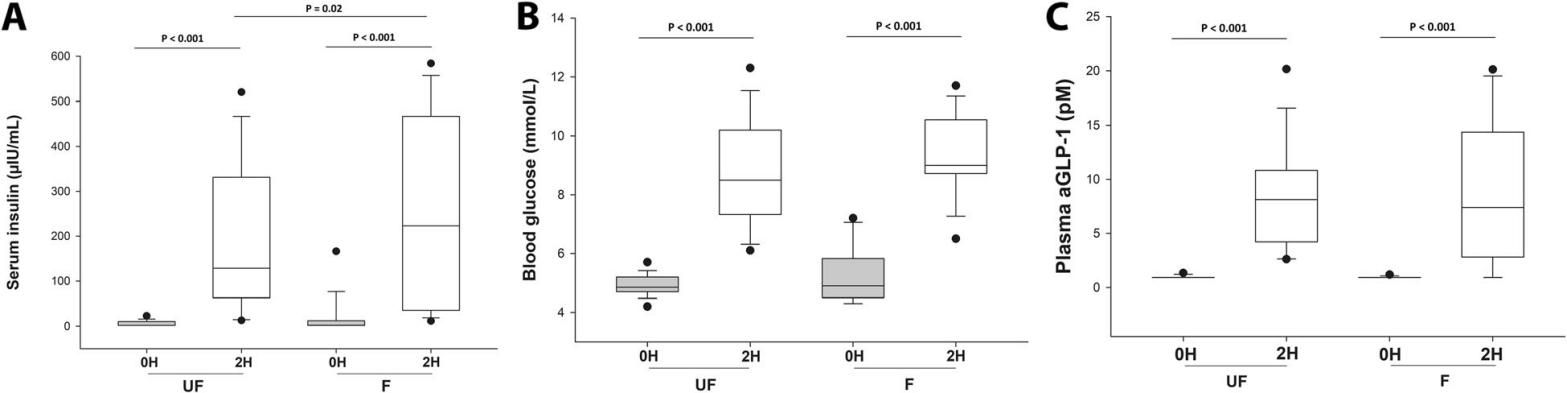
Basal and post-prandial hormone concentrations measured during an oral glucose test in ponies after grazing. Hormone concentrations were measured before (0H) and after (2H) a test meal during an oral glucose test in 16 ponies after grazing either unfertilised (UF), or fertilised (F), pasture for 4 h/day for 5 days (with an evening meal of hay). The post-prandial serum insulin (**a**) and blood glucose (**b**) concentrations were higher than the resting concentration after both diets, and insulin was higher after eating F pasture, compared to UF pasture. The plasma aGLP-1 concentration (**c**) was higher than the resting concentration after both diets, but did not differ after each grazing phase

As expected, the post-prandial serum insulin concentration was higher (*P* < 0.001) than the resting (0 h) concentration for both experimental OGTs (Fig. 1a). The mean resting (0 h) serum insulin concentration was similar (*P* = 0.36) after 5 days of grazing fertilised, compared to unfertilised grass (Fig. 1a). However, the post-prandial serum insulin concentration was higher (*P* = 0.018, two-tailed) after 5 days of access to fertilised pasture, compared to accessing unfertilised pasture (Fig. 1a).

Similarly, while the post-prandial blood glucose concentration was higher (*P* < 0.001) than the resting (0 h) concentration for both OGTs, the mean resting (0 h) glucose concentration was not affected by study phase (*P* = 0.93; Fig. 1b). However, unlike insulin, the post-prandial blood glucose was not higher (*P* = 0.34, two-tailed) after 5 days of access to fertilised pasture, compared to unfertilised pasture (Fig. 1b). The plasma concentration of the incretin hormone aGLP-1 also increased after eating both pastures (*P* < 0.001; Fig. 1c), but the resting concentrations were barely detectable, and were therefore not different between phases. The post-prandial aGLP-1 concentration was similar after access to unfertilised, and fertilised, pasture (Fig. 1c, *P* = 0.78). Post-prandial plasma GIP similarly did not differ after access to unfertilised (179 ± 62.2 pg/mL), or fertilised (185 ± 82.5 pg/mL), pasture (*P* = 0.82).

#### Sub-group responses

**Fig. 2:**
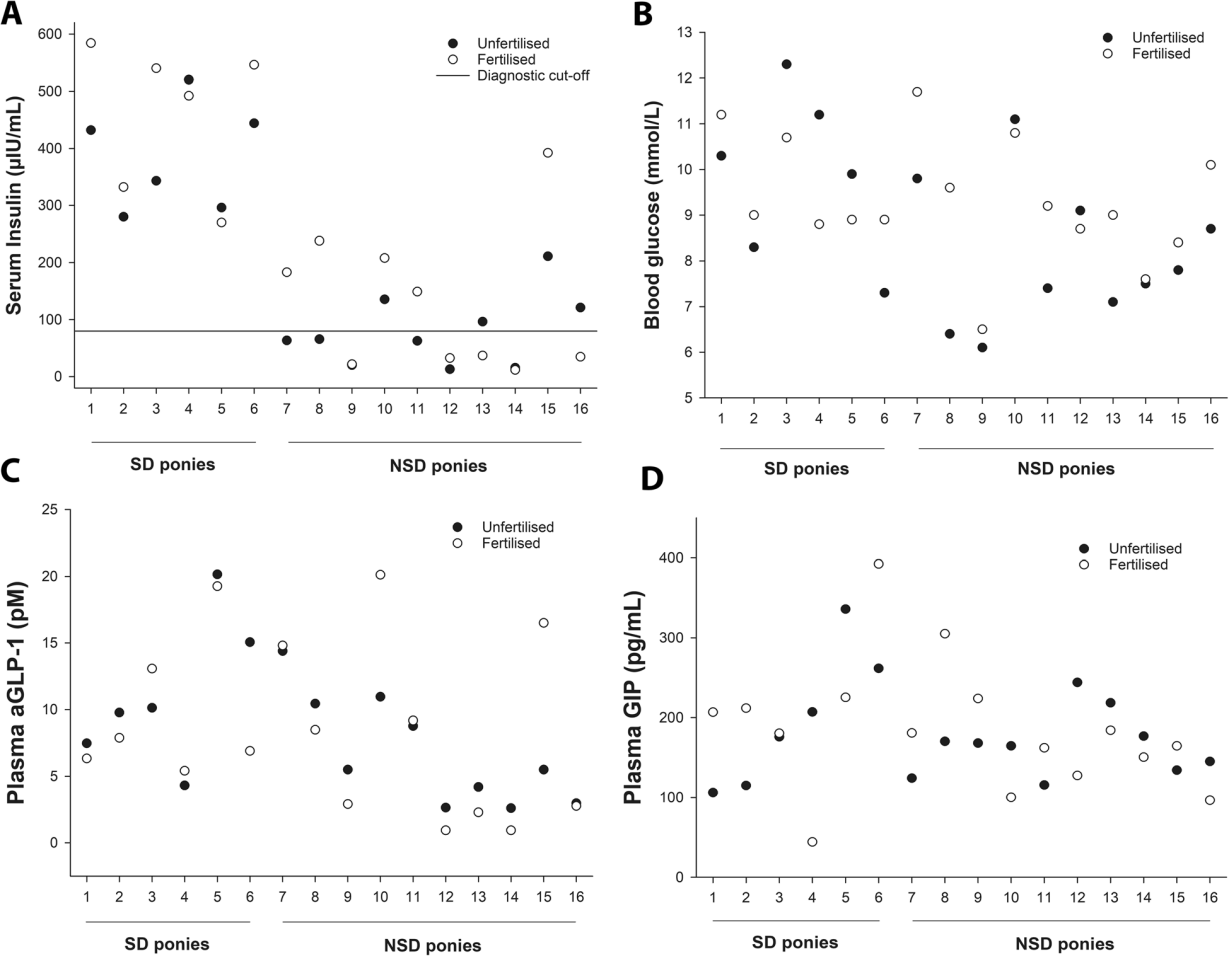
Post-prandial hormone concentrations during an oral glucose test in insulin-dysregulated ponies. The hormone concentrations were measured in16 ponies previously determined to be either severely insulin-dysregulated (SD) or not severely insulin-dysregulated (NSD). Measurements were taken after grazing either unfertilised (●), or fertilised (○), pasture for 4 h/day for 5 days (with an evening meal of hay). The insulin responses to each test (**a**) were lower, but more variable, in NSD ponies. While the SD group were consistently insulin-dysregulated across both tests, the NSD responses were more variable, and were sometimes above the OGT test cut-off (□) and sometimes below it. The blood glucose (**b**) plasma aGLP-1 (**c**) and plasma GIP (**d**) did not differ between groups after grazing either pasture and the variability across the two tests was similar

Grouping the ponies as NSD or SD revealed some outcomes that were not apparent when the data were analysed as a single cohort. In line with the initial OGT, the post-prandial insulin response to the experimental OGTs was markedly higher in SD ponies, compared to NSD ponies, after grazing both unfertilised (386 ± 94 μIU/mL and 80 ± 63 μIU/mL, respectively) and fertilised (461 ± 129 μIU/mL and 131 ± 126 μIU/mL, respectively) pasture (*P* < 0.001). Further, the post-prandial OGT insulin concentration never fell below 270 μIU/mL in any pony in the SD group after either pasture, and the insulin response was less variable (CV: 15 ± 4.15%), compared to the NSD group (CV: 50.6 ± 8%, Fig. 2a). Examination of Fig. 2a reveals that the SD group were consistently insulin-dysregulated across both tests, while the NSD group exhibited more variable responses, and were sometimes above the OGT test cut-off of 80 μIU/mL, and sometimes below it. In half of the NSD ponies, a diagnosis of ID would have been made after one phase, but not the other.

The blood glucose, plasma aGLP-1 and plasma GIP did not differ between groups after grazing either pasture. The variability in post-prandial blood glucose concentration (SD 10.0 ± 4.59% and NSD 9.9 ± 8.57%; Fig. 2b), post-prandial plasma aGLP-1 (SD 19.4 ± 17% and NSD 35.6 ± 27.7%; Fig. 2c) and post-prandial plasma GIP (SD 39.5 ± 29.8% and NSD 25.5 ± 11.5%; Fig. 2d) was similar after the two tests.

#### Relationships between variables

**Fig. 3:**
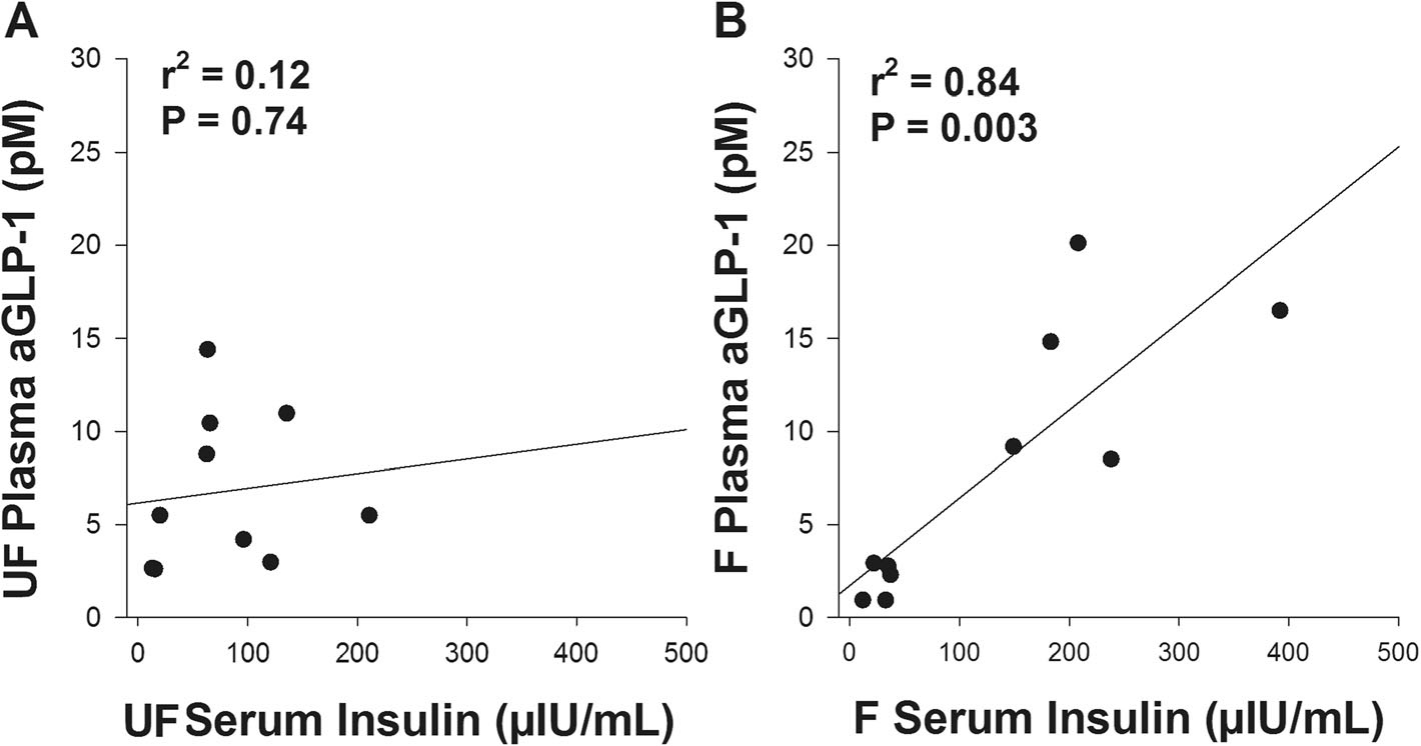
The relationship between insulin and incretin responses to an oral glucose test during grazing. Serum insulin and plasma aGLP-1 responses to an OGT after 5 days of eating a diet of hay/unfertilised pasture (UF) and hay/fertilised pasture (F) in not severely insulin-dysregulated ponies (NSD; *n* = 10). The two hormones were not correlated in NSD ponies after eating hay/unfertilised pasture (**a**), but were after hay/fertilised pasture (**b**)

For the entire cohort, no significant linear correlations were observed between post-prandial insulin and the potential predictor variables of glucose and GIP. However, a linear relationship was found with aGLP-1. Therefore, plots of the correlation data for aGLP-1 and insulin were examined in detail, by considering the NSD and SD cohorts separately (Fig. 3). This revealed that there was no correlation between aGLP-1 and insulin for SD ponies on any of the diets (hay/unfertilised, r^2^ = − 0.6, *P* = 0.3; hay/fertilised, r^2^ = − 0.6, *P* = 0.2), whereas for the NSD ponies, there was an apparent positive correlation following the hay/fertilised pasture diet (Fig. 3b).

Further modelling of the variability of the insulin was performed, with the final model including an interaction term between diagnostic classification status and 2 h post-prandial aGLP-1, with a final AIC value of 49 and normally distributed residuals. A previous diagnosis of being SD was the best predictor of a hyper-physiologic insulin response to the OGT (estimate ±95%CI; 1.2 ± 0.6–1.8; *P* < 0.001), with those previously diagnosed as NSD having a lower response than the SD group. The post-prandial insulin concentration of the NSD group increased (0.05 ± 0.02–0.08; *P* = 0.002) in association with increases in aGLP-1 within each phase The effect of aGLP-1 in the SD group was significantly lower than in the NSD group (− 0.07 ± − 0.12 – -0.018; *P* = 0.01) which resulted in no augmentation of insulin due to aGLP-1 in the SD group.

## Discussion

This study has demonstrated that forage diets containing a combination of hay and fresh pasture can differentially impact metabolic responses in ponies. Pasture grazing is a known risk factor for endocrinopathic laminitis [3], and therefore studies on the metabolic responses to grazing are important. Currently, few studies have examined the effect of pasture grazing on the insulin response in horses and ponies, especially those that are SD. This study reports acute changes in the response to an OGT following grazing fertilised pasture, compared to unfertilised pasture, in ponies. Also of interest was the finding that mild or early ID may only become apparent when feed quality increases and/or availability is increased, with a potential role for aGLP-1 in augmenting insulin responsiveness in these ponies [21].

Pasture fertilisation greatly increased pasture availability in the current study. This is consistent with agronomy studies where treating pastures with a nitrogen-based fertiliser increased the growth rate over the spring season when rainfall and temperature were increased [11, 13]. Further, although nitrogen fertilisation of timothy grass did not affect the NSC content, it did result in a higher dry matter yield [12, 25]. In the current study, the fertilised pasture yielded an increased herbage volume, without a change in NSC content. It has been shown previously that fertilised pasture is likely to be lower in NSC content, compared to unfertilised pasture [26], and reasons why this did not occur in the current study could be due to grass type, environmental conditions or the duration of the study. The greater availability of pasture on the fertilised strips (both the height and density of the grass) likely enabled greater pasture intake by the ponies, compared to grazing unfertilised pasture, but this variable was not successfully measured. It is feasible that greater pasture intake on fertilised pasture contributed to the higher post-prandial insulin response to the OGT after grazing fertilised pasture. The failure to estimate pasture intake was a weakness of the study, and future investigations should aim to interrogate a potential relationship between intake and metabolic responses by quantifying pasture intake. Studies on pasture intake/availability and NSC content would be valuable because if increased pasture intake does occur on fertilised pasture (or any pasture with greater herbage mass) then access to lush pasture should be prevented or carefully controlled in ponies, particularly those with ID.

Pasture restriction is a recommended management tool for ponies diagnosed with ID [27]. This can been achieved by utilising grazing muzzles or dry yards [27]. The current study utilised restricted grazing times of 4 h/day and dry yards to help limit pasture intake. Further, all of the dietary forages used had an NSC below 10%, which is the current recommendation for feeding ponies at risk of ID and laminitis. However, only three ponies from the NSD group were consistently below the diagnostic cut-off of 80 μIU/mL of insulin when tested with an OGT after both grazing phases. This is despite all of the NSD ponies being below this cut-off prior to the study. This outcome suggests that 1) the OGT is not very precise in some animals, or 2) that animals with mild or early ID undergoing dietary modification are more metabolically unstable, or that a combination of both factors may be involved. We suggest that ponies undergoing a change in diet composition need to be carefully monitored for ID, and that their risk of post-prandial hyperinsulinaemia can potentially be managed with a dietary change.

Our data also showed that where moderate access to pasture unmasked latent/early ID in some ponies, that augmentation of insulin secretion in these ponies might have been driven more by aGLP-1, than occurs in animals with more severe ID [28]. While aGLP-1 concentrations have been shown to be higher in ponies with ID compared to healthy ponies [21], horses with equine metabolic syndrome that were fed a high NSC diet for 8 weeks also did not show changes in their aGLP-1 response to an OGT [29]. Therefore, another mechanism, such as altered glucose bioavailability, may be involved once marked ID becomes apparent [21]. However, if aGLP-1 plays a more central role in early ID, monitoring changes in this hormone may be beneficial when diagnosing the condition, and further studies are warranted. In addition, GLP-1 receptor antagonists have been reported as a potential (partial) treatment for equine ID [30], and may be a useful modality for reducing insulin augmentation in ponies with mild or early disease.

Regardless of the underlying pathophysiology of ID, our results do indicate superior repeatability of the OGT in SD ponies, with a diagnosis of ID much more likely to be made consistently in this group. Further, it has been previously suggested that the threshold for an increased risk of laminitis is a serum insulin concentration of approximately 200 μIU/mL in both horses [31], and ponies [24]. At this threshold, all of the SD group would have been at increased risk of laminitis while grazing for 4 h a day, regardless of the quantity of grass available. Thus, dietary restriction alone might not be sufficient for the management of ID in these animals and the development of other therapeutic options, such as novel drugs, needs to be prioritised.

By comparison, the results of the current study challenge the precision of the diagnostic outcome of the OGT in ponies with mild or early ID. Our results also suggest that a change in diet might impact the insulin response to an OGT in some ponies (when fasted prior to the test). The current data suggest that pasture quantity is important, but insulin responses were shown to be greater after feeding a hay higher in NSC (compared to a low NSC hay) to healthy horses, highlighting that NSC is still an important consideration during diet formulation and the management of ID [32]. Thus, assessing the different types of forages in the diet, including quality and availability, is likely to be important when utilising the OGT for diagnostic purposes, particularly if the animal’s metabolic health is to be monitored over time with repeated OGTs. Taken together, these data also suggest that the OGT could potentially be used to monitor the effectiveness of dietary management strategies in animals with ID, but this requires specific investigation.

## Conclusions

This study demonstrated that changes in the forage component of a diet can affect the responses to an OGT in ponies with mild ID, and that this could be driven by aGLP-1. Dietary management is therefore likely to be a useful method of controlling ID in these ponies. However, ponies with more severe ID may require other interventions, such as medications, to prevent prolonged hyperinsulinaemia and reduce laminitis risk. The OGT is a useful tool for assessing ID. However, the current feeding procedures need to be considered when using the test, especially in ponies that are suspected to be mildly, or latently, insulin-dysregulated.

## Methods

### Animals

Animal work was approved by the Animal Ethics Committee of The University of Queensland (QUT/SVS/316/16). Sixteen mixed-breed ponies (6 x Shetland/Shetland cross-breed, 3 x Welsh/Welsh cross-breed, 7 x other breeds) owned by Queensland University of Technology were used. Their body condition score (BCS) was assessed on the Henneke [33] scale of 1 (very poor) to 9 (very fat), and their cresty neck score (CNS) was determined as described by Carter et al. [34]. All morphometric measurements were undertaken by an experienced assessor. Haematology, biochemistry and ACTH test results were all within acceptable ranges (ACTH values were interpreted according to season and location i.e. Southern Hemisphere [35]). The ponies were sold at the conclusion of the study.

### Study design

For the 2 weeks prior to the study the ponies were housed in small groups (2–5 individuals) in bare paddocks and fed a grass hay at 1.8–2% BW (divided into 2 meals given in the morning and evening). During this time the ponies were tested for insulin responsiveness using a standard OGT. Briefly, all ponies were fasted overnight prior to consuming a diet that consisted of 1 g/kg BW dextrose, 200 g wheat bran, 0.3% BW lucerne chaff. Resting (0 h) and post-prandial (2 h after the meal) blood samples were collected. All ponies were classified based on their post-prandial insulin concentration during this test, prior to entering the study. Ponies were grouped as either NSD (< 80 μIU/mL) or SD (≥ 80 μIU/mL) based on a published cut-off value for post-prandial serum insulin during this test [19, 36]. Although a post-prandial serum insulin concentration of < 80 μIU/mL could be considered healthy when using the OGT as a diagnostic test with a binary outcome, it became apparent during the study that some ponies in this group were not metabolically normal, hence the use of the term NSD.

At study commencement the ponies were moved into individual dry lot accommodation and fed a 2% BW lucerne hay diet (sourced from a single batch), plus a commercial balancer pellet fed at 0.15 g/kg bodyweight (Gold Pellet, Kentucky Equine Research). During the study all ponies were fed this hay diet for the first 10 days (prior to the first grazing phase), and during the interim 10-day period between the grazing phases (Table 3). The study timeframe ensured that the study was completed within 1 month, which aided in minimising any seasonal effect on the pasture. The study was undertaken as a randomised, repeated measures design (Table 3), so half of the ponies were randomly allocated to graze unfertilised pasture in the first phase, while the other half grazed adjacent pasture that was of identical composition, but had been fertilised for study purposes. The grazing area, which contained a pasture mix of couch, rye grass and clover, was halved and a 5 m wide pathway was placed down the middle to enable easy access to each strip, and also to accommodate any fertiliser run-off. Half of the study site was fertilised before the study (at the start of the growing season) and again lightly 3 days before each grazing phase (irrigated in) with a commercially available fertiliser (CK88, Incitec Pivot Fertilisers). The other half remained unfertilised, with both sides irrigated equally.

**Table 3:** The randomized, cross-over study design for two groups of ponies grazing fertilised and unfertilised pasture

Phase	Hay-only	Grazing	Hay-only	Grazing
No. of days	10	5	10	5
Lucerne hay (2% BW) in dry-lots	X		X	
Fertilised pasture 4 h + lucerne hay (0.7% BW)		Group 1		Group 2
Unfertilised pasture 4 h + lucerne hay (0.7% BW)		Group 2		Group 1
Oral glucose test (morning after end of phase)		X		X

Key: 8 ponies were allocated to each group

The ponies were let out to graze the individual strips (4.2 m × 21 m) of grass between 8 am - 12 pm (4 h) for 5 consecutive days, before returning to their individual dry lot. The evening meal comprised 0.7% BW lucerne hay, plus a commercial balancer pellet as above. Faeces was removed from the grazing area, and the dry lots, on a daily basis. An OGT, using a lower dose (0.75 g/kg BW) of dextrose [19] but the same sampling regime, was performed on each pony the day after each grazing period (Table 3). The lower dose of dextrose was selected for use during the experimental phase because of improved palatability and speed of consumption, without compromise to the magnitude of the insulin response, as previously observed [19]. Attempts to estimate pasture intake were made by weighing the ponies before and after pasture access each day, but measurement of this variable was adversely affected by body water loss (associated with high ambient temperatures) and the data are not reported.

### Samples

The pasture was sampled at 10 am on the day prior to each grazing period. Five 10 × 10 cm plots were randomly selected, using a backwards-thrown setsquare, and were sampled to a level 1 cm above the ground. These samples were pooled, mixed and immediately dried using a microwave oven. The dry matter was calculated before ‘grab’ sub-samples were sent to a commercial laboratory (Department of Primary Industries, Australia) for further analyses. The lucerne hay was randomly sub-sampled from 10 bales using a bale corer and analysed at the same commercial laboratory.

During each OGT, blood glucose was measured immediately in whole blood using a hand-held glucometer (Accu-Check, Roche Diagnostics) previously validated for equine blood by the investigators. The remaining blood was separated between clot activator (serum), and DPP4 inhibitor-containing (plasma) vacutainer tubes (Becton Dickson). Clot activator tubes were left to clot at room temperature for 30 min, centrifuged (1500 g × 10 min) and serum collected and stored at − 20 °C. The DPP4 inhibitor tubes were immediately put on ice for 10 min, centrifuged (1500 x *g* × 10 min) and plasma collected and stored at − 20 °C. Samples were transferred to − 80 °C storage at the end of each phase, prior to analyses. The serum insulin concentration was measured at a commercial laboratory using a chemiluminescent assay (Vetpath, Australia). The plasma concentrations of the incretin hormones, aGLP-1 (intra-assay CV 3.4% and inter-assay CV 13.8%) and GIP (intra-assay CV 4.4% and inter-assay CV 8.5%), were measured using previously validated ELISA kits (EZGLPHS-35 K and EZHGIP-54 K respectively; Merck Millipore) [21, 37].

### Statistical analyses

Fodder, hormone and pony morphometric data analyses were performed in SigmaPlot v.13 with a significance level of *P* < 0.05 accepted. Data are reported as mean ± sd or median (interquartile range). The data were tested for normality using the Shapiro-Wilk test. The unfertilised and fertilised pasture (dry matter, sward height and sward density) were compared using a t-test. The pre- and post-prandial hormonal variables were analysed using a paired t-test, or Wilcoxon Signed-Rank test if the data were not normally distributed. The variability of repeated measures was assessed with co-efficient of variation (CV). Associations between post-grazing OGT glucose, insulin, aGLP-1 and GIP were examined using Pearson’s Correlation test. Comparisons between SD and NSD pony groups were made using a t-test, or the Mann-Whitney Rank Sum Test when data were not normally distributed.

The insulin concentration was modelled with a linear, mixed-effects model using the statistical programme SPSS (IBM V23). The 2 h post-prandial serum insulin concentration was log_10_ transformed to achieve a normal distribution of the residuals. The model included the repeated effect of phase on each pony, and used a compound symmetry covariance structure. Akaike’s Information Criterion (AIC) was used to assess model fit between iterations [38]. The log_10_ post-prandial serum insulin concentration was used as the dependent variable, with phase; diagnostic classification status; 2 h post-prandial glucose; 2 h post-prandial aGLP-1 and 2 h post-prandial GIP as fixed factors, and individual pony as a random effect.

## Supplementary information

**Additional file 1.** Raw data file.

## Data Availability

All data generated or analysed during this study are included in this published article [and its Additional file 1].

## Abbreviations

ACTH: Adrenocorticotropic hormone
aGLP-1: Active glucagon like peptide-1
BCS: Body condition score
CNS: Cresty neck score
GIP: Glucose-dependent insulinotropic polypeptide
ID: Insulin dysregulation
NSC: Non-structural carbohydrate
NSD: Not severely insulin dysregulated
OGT: Oral glucose test
SD: Severely insulin dysregulated
WSC: Water soluble carbohydrate
